# Bridging the gap between human behaviour and animal welfare: A study on human behaviour change and body condition scoring of suckler cows

**DOI:** 10.1017/awf.2026.10072

**Published:** 2026-03-03

**Authors:** Lesley Jessiman, Cynthia Joanne Naydani, Kenneth MD Rutherford, Simon P Turner

**Affiliations:** 1 Animal Behaviour and Welfare, School of Veterinary Medicine & Biosciences, Scotland’s Rural College, Edinburgh, UK; 2 The University of Edinburgh Royal Dick School of Veterinary Studies, Roslin, Midlothian, UK

**Keywords:** Animal welfare, Behaviour Change Wheel, cows, human behaviour change, stockperson, suckler beef cattle

## Abstract

There are several examples of best animal husbandry practices that are not adopted, leading to animal welfare compromises. Bridging this gap between advice and human behaviour is crucial in helping drive improvements in animal welfare. Inappropriate feeding of pregnant cows is common and associated with compromised health and welfare. Obesity and leanness can cause calving difficulty and reduce the vigour of newborn calves. One way to offset the problems associated with body condition extremes is to adopt body condition scoring (BCS) by hand. Knowing each animal’s condition helps the farmer identify ‘at risk’ cows leading to better feeding decisions and improved health and welfare. Despite the significant benefits of BCS, very few farmers routinely adopt this practice, relying more upon a visual assessment of condition. Some farmers also report that they do not BCS by hand, or by eye. The current study identified the key barriers and drivers of BCS by hand to develop an evidence-based intervention designed to encourage more adoption. We propose that human behaviour change frameworks, such as the Behaviour Change Wheel (BCW), present the opportunity to address other animal welfare issues where best management practices are rarely adopted. We also recommend that an interdisciplinary team of animal welfare and social scientists are best positioned to develop human behaviour change interventions that will more likely lead to tangible, persistent and positive change.

## Introduction

Excessive leanness or obesity in pregnant animals can have undesirable health and welfare outcomes both for the dam and for developing fetal progeny. In cattle, extreme leanness can contribute to calving difficulties, which is associated with increased pain, trauma and associated disease risk, exhaustion and increased need for stockperson handling. Poor condition and calving difficulty can also result in poor calf development and/or subsequent failure of the mother to conceive again (Barrier *et al.* 2012; Diskin & Kenny 2016; Bragg *et al.* 2021; Fordyce *et al.* 2021). Obesity may also contribute to calving difficulty and additionally represents inefficient use of feed resources causing undue financial costs. There are environmental ramifications associated with feed waste (Reynolds *et al*. 2011; Drackley & Reynolds 2021), and prior research has shown a strong positive correlation between dry matter intake and methane production (Herd *et al.* 2014). Providing appropriate feed quantity and quality to maintain body fat within an acceptable range is therefore expected to deliver multiple benefits. However, a recent study of commercial beef farms in the UK found that 20% of pregnant cows were too lean prior to spring calving (Rutherford *et al.* in prep). Collation of data from multiple beef units in New Zealand also showed substantial variation in cow body condition both within the annual production cycle and between farms (Weik *et al.* 2022). Research has also shown that instances of obesity in dairy cattle parallel those observed in the human population (De Koster & Opsomer 2012), with over-conditioning resulting in significant health issues (Guo *et al.* 2024).

The recommended approach for monitoring fat deposits in a practical context is body condition scoring (BCS), which requires physical palpation of fat deposits by hand to provide a subjective judgement of subcutaneous fat depth at key points on the body (Lowman *et al*. 1976). Animals are then assigned a value on a categorical scale. BCS can be conducted quickly, with minimal training, when performed alongside other routine animal handling tasks (e.g. vaccinations and pregnancy testing). Despite the apparent ease of BCS and the benefits of maintaining body condition within an acceptable range, only 4% of UK beef farmers use the recommended approach of hands-on assessment (SAC Commercial 2013). Whilst most (61%) farmers reported that they judged body condition visually, from a distance, variation in coat depth and uterine expansion during pregnancy can alter the apparent fat depth visible by eye (SAC Commercial 2013). Therefore, judgement by visual inspection is likely to be less effective at detecting animals close to the borderlines of the acceptable body condition range compared to BCS scoring by hand.

In the UK, the recommended hands-on approach to BCS and the associated scoring scale have been advocated for 50 years (Lowman *et al*. 1976). The low uptake contrasted against the expected benefits of optimum body condition management suggest an ingrained reluctance to adopting this evidence-based approach. The aim of this study is therefore to draw upon behavioural science to better understand the barriers responsible for this suboptimal uptake of BCS scoring by hand and to develop a potential human behaviour change intervention that would encourage the adoption of this target behaviour.

Several studies have drawn upon social and/or behavioural science to examine the influences on farmers’ behaviours and cognitions in relation to animal welfare (Balzani & Hanlon 2020). Specifically, research has focused on the role that attitudes (Bock & Van Huik 2007; Kauppinen *et al.* 2013; Coleman & Hemsworth 2014), empathy (Kielland *et al.* 2010; Peden *et al.* 2020; Browne *et al.* 2022) and motivations (Valeeva *et al.* 2007; Hansson *et al.* 2018) play in relation to animal welfare decisions and practices. Several studies have also looked at attitudes and behaviours through a social cognitive lens, namely the Theory of Planned Behaviour (de Lauwere *et al.* 2012; Winkel *et al.* 2020), which proposes that behaviours stem from appraisal of social norms, attitudes and intentions (Ajzen 1985). Although a social cognitive lens can offer valuable insights into the influences on farmers’ behaviours, it can sometimes be too focused on the individual; failing to fully account for how individual factors are shaped by, and interact with, the broader social, economic, historical, and/or cultural contexts.

Given the complex nature of farming, farmers, and animal welfare-related issues, it is important that we fully understand all the influences of a behaviour, before we attempt to change it. More recently, researchers have looked beyond the individual-focused theories, models and frameworks, drawing directly from the Behaviour Change Wheel (BCW: Michie *et al*. 2014) to identify the key drivers and barriers of animal welfare-related behaviours. The BCW is a comprehensive theoretical model that considers human behaviour as a complex and interactive system, looking at both the individual and the environments in which the individual interacts (Michie *et al*. 2014). Animal welfare researchers have therefore turned to the BCW to bridge the gap between what evidence suggests would improve animal welfare standards and the real-world implementation of that information. For example, several researchers have identified the many ways in which adoption of the BCW model can increase the likelihood of successfully reducing tail biting in pigs (Carroll & Groarke 2019; Carroll *et al.* 2021), increase the longevity of cat neutering campaigns that promote cat welfare (McDonald *et al.* 2018), improve the husbandry of small mammal pets (Carroll *et al.* 2025), reduce sheep and cattle lameness (Clark *et al.* 2024), tackle the equine obesity problem (Furtado *et al*. 2022) and increase responsible antibiotic prescription on dairy farms (Farrell *et al.* 2023).

**Figure 1.**
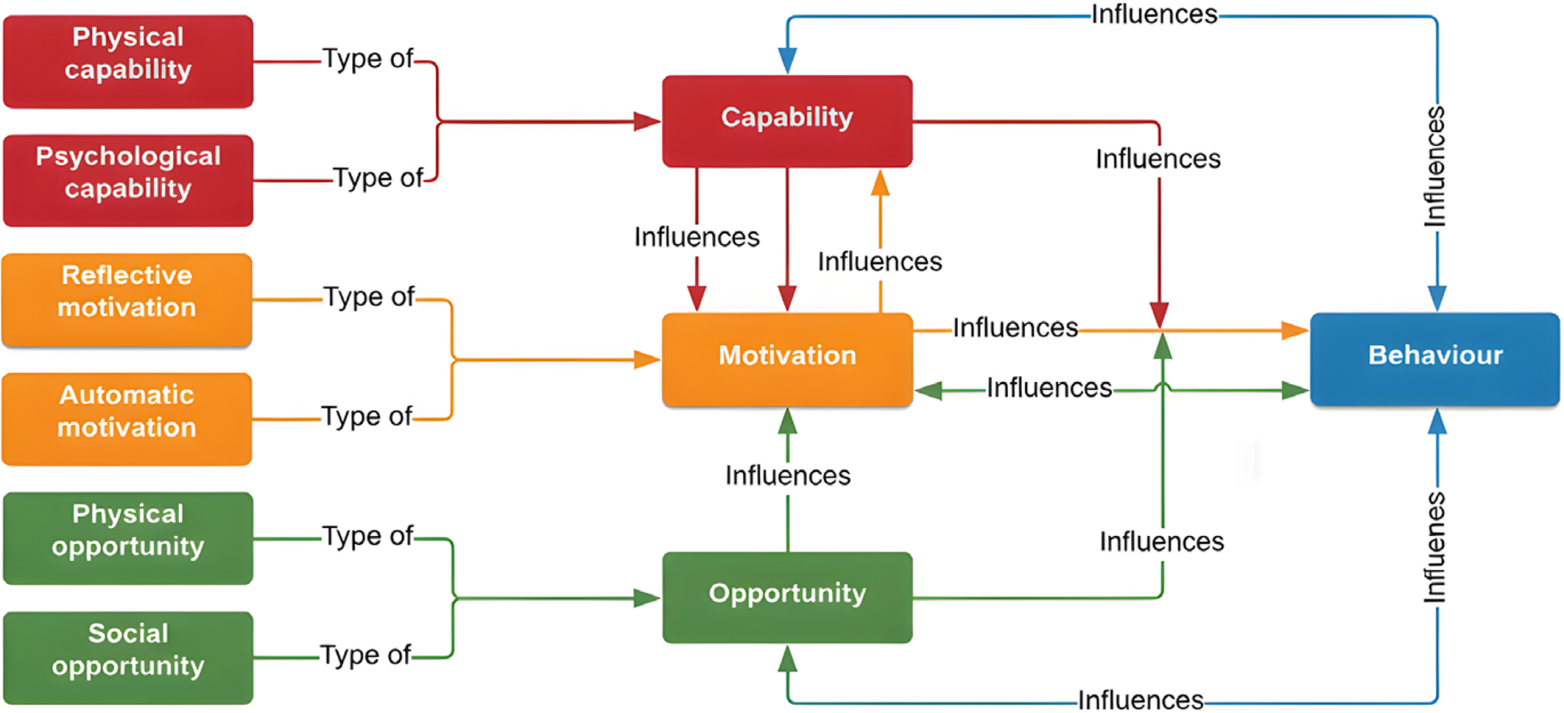
The Capability-Opportunity-Motivation-Behaviour (COM-B) Model of Behaviour Change, reproduced with permission from West and Michie (2020).

At the core of the BCW is the capability-opportunity-motivation-behaviour (COM-B) model (Figure 1), where the components of Capability (psychological or physical), Opportunity (social or physical), and Motivation (automatic or reflective) are considered as influencers of Behaviour (Michie *et al*. 2014). Unlike other models of behaviour change, the COM-B also considers the importance of context (West & Michie 2020). For a behaviour to occur one must have the capability and opportunity with which to engage in that behaviour, with the motivation to engage in the desired behaviour exceeding the motivation for competing behaviours (Michie *et al*. 2014; West & Michie 2020). Changing a behaviour will therefore involve changing one or more of the influences of that behaviour, placing the COM-B system into a new configuration and reducing the likelihood of the individual reverting back to the undesired behaviour (Michie *et al*. 2014; West & Michie 2020).

The COM-B model is underpinned by the Theoretical Domains Framework (TDF: Michie *et al.* 2005; Cane *et al*. 2012). The TDF, which has been subject to rigorous validation, provides theoretical insights into the specific cognitive, affective, social, and environmental influences on human behaviour (Cane *et al.* 2012). The TDF therefore provides a more granular account of behaviour than the COM-B. For example, the COM-B diagnosis may reveal ‘reflective motivation’ is a key influence of behaviour, but the TDF can also tell us the specific construct involved, e.g. self-efficacy or perceived behavioural control (Atkins *et al.* 2017).

Although the BCW has been adopted by several other animal welfare-focused studies (McDonald *et al.* 2018; Carroll *et al.* 2021; Clark *et al.* 2024) as far as we are aware, this is the first study to use the BCW for our target behaviour (i.e. adoption of BCS by hand) and with our target audience (i.e. Scottish suckler cattle farmers). Additionally, several studies have used the BCW to map the influences of behaviour onto the COM-B model and/or the TDF (Clarke *et al.* 2024) but far fewer studies have engaged in a full intervention design process, stopping short of identifying the Behaviour Change Techniques or developing a potential intervention. As encouraged by Carroll and Groarke (2019), a full intervention design is necessary to change behaviours that will lead to positive and lasting animal welfare outcomes. Our principal aim in the current study is therefore to go beyond COM-B and TDF mapping to create a rigorous and evidence-based intervention that can later be implemented and tested. Knowing how and why an intervention was designed, i.e. identifying the theoretical framework, the interventions, the policy categories and the behaviour change techniques, is key to successful assessment and/or replication of an intervention (Cotterill *et al.* 2018; Shahsavari *et al.* 2020).

## Materials and methods

### Ethical considerations

This study was approved by the Scotland’s Rural College’s Social Science Ethics Committee (protocol#116/73026312). All participants were provided with a detailed participant information document that outlined the key ethical considerations, such as procedure, withdrawal, confidentiality/anonymity and data storage. All participants provided written or verbal consent for their data to be used for the purposes of this study.

### Study design

This study employed an intervention development design, guided by the Behaviour Change Wheel framework. The design employed qualitative methods (i.e. focus groups) to identify barriers and drivers of BCS by hand. The design also included an inter-rater reliability analysis, which was applied during the systematic mapping of barriers to COM-B components and TDF domains/constructs to ensure rigour and consistency in the intervention design process.

### Participants

Data were collected from six focus groups, consisting of 10 to 15 participants in each group (n = 72). All of the farmers that participated in our focus groups had the opportunity to complete a questionnaire, which consisted of some background questions and questions/statements regarding their BCS practices. Of those farmers who completed the participant age section of the questionnaire (n = 23), there were two young adults (ages 18–44), 15 middle-aged adults (ages 45–59), and six older adults (> 60 years). All 72 farmers that participated in the focus groups lived in Scotland (three different regions) and had herds that ranged from 4 to 480 cows. The farms ranged from lowland, upland and hill. We should note that we did not collect background data as analytical categories but simply to illustrate who we spoke with in our study.

### Materials and apparatus

A semi-structured interview guide was used to generate discussions around BCS by hand. The semi-structured interview guide consisted of the following questions and prompts, for example:

1. *Can we begin with just seeing how many of you body condition score your cattle; Follow on: do any of you get your hands on? Prompt: can you tell me why you get your hands on/why you don’t get your hands on?*
2. *What about recording scores, do any of you formally record your body condition scores? Prompt: can you tell me why you record your scores/why you don’t record your scores?*
3. *Does anyone speak to their vet about the condition of your cows? Prompt: can you tell us a little more about that; do they bring it up; do you bring it up?*
4. *Does anyone speak to anyone else, e.g. a nutritionist about condition? (all followed by prompt: why, why not?) and*
5. *Has anyone ever had any issues, such as cows being over or underweight pre-calving? Can you elaborate?*

We should note that the semi-structured interview guide was not designed around the COM-B model. COM-B mapping only occurred post-data collection after the categories and themes had been abstracted from the focus group transcripts. We chose not to use the COM-B to design our questions as we wished to identify the barriers and drivers of BCS adoption in the absence of researcher-imposed theoretical categories or frameworks.

A short questionnaire was also used which consisted of background and husbandry questions or Likert scale statements about the farmers’ adoption of BCS (e.g. *please provide your level of agreement/disagreement with the following statement:* “Body condition scoring does little to improve cow welfare”). We also asked open-ended questions about BCS practices (i.e. (1) *please tell us any reasons you feel body condition scoring can be challenging or is not necessary* and (2) *Please tell us about any other benefits you feel that body condition scoring can bring if these have not been covered in this questionnaire).* It was only the answers to the open-ended questions that we wished to draw from for the purposes of this current study. The other questions/statements were designed for a different study. Copies of the focus group interview schedule and the full questionnaire can be found in File A (see Supplementary material).

### Procedure

Focus groups occurred at various farming knowledge exchange events from December 2022–June 2023. One of the focus groups followed an off-farm afternoon of technical talks and industry updates, one followed an on-farm open day and a presentation of the host farmer’s system, and four took place as part of an on-farm meeting in which farmers rotated in small groups around five technical presentations. Focus groups were moderated by ST, who is an animal welfare scientist. As such, we should note that it is possible that there was a perceived power dynamic between the participants and ST that may have influenced the participants’ responses.

Discussions lasted from 25–40 min, which we acknowledge is shorter than most focus group sessions. However, given our aims, the discussions were very much on-point. The length of the discussions was also directed by the participants in each of the focus groups. The seating of the focus groups formed a wide U-shape around ST who facilitated the discussion sessions. ST adopted techniques to ensure everyone had the opportunity to speak.

At the end of the focus group sessions, we also disseminated a hard copy questionnaire. The responses to the two open-ended questions were content analysed alongside the focus group transcripts.

All the focus group sessions were recorded using a high-resolution WAVE/MP3 recorder (R-09HP by Roland Corporation, Shizuoka, Japan). Transcriptions were produced using Otter Ai. The focus groups were recorded, transcribed and personal identifiers removed to ensure anonymity.

### Content analysis and BCW mapping procedures

**Figure 2.**
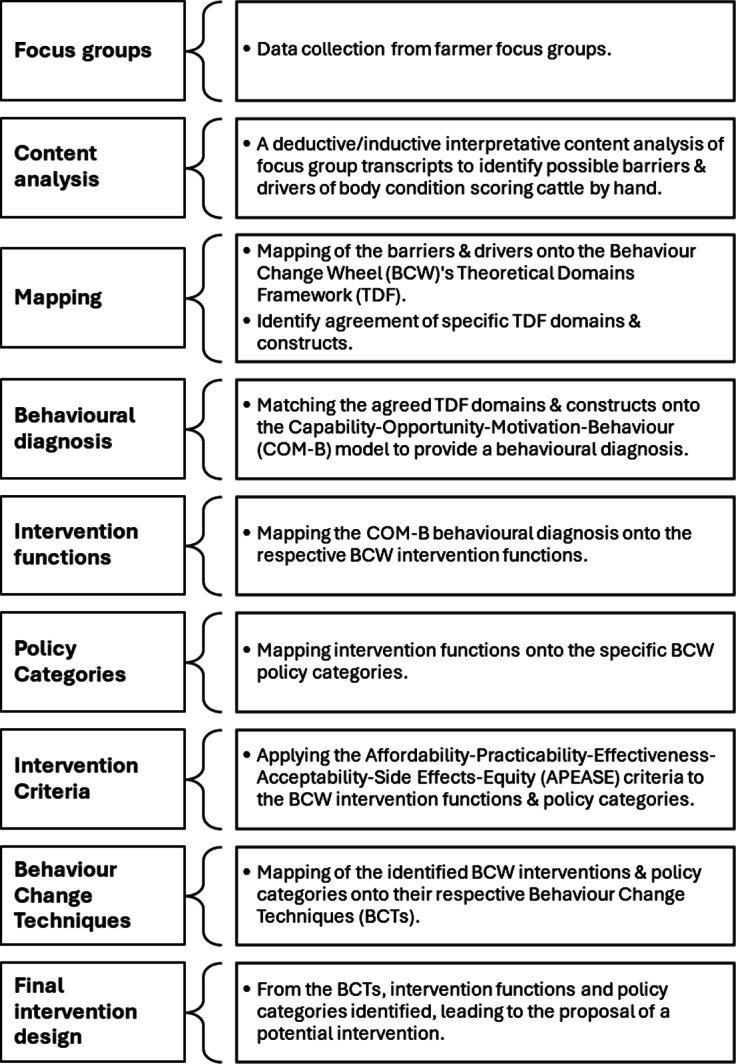
A step-by-step summary of the research process adopted by the authors for developing a human behaviour change intervention using Michie *et al.*’s (2014) Behaviour Change Wheel. This process aimed to identify barriers towards the uptake of Body Condition Scoring (BCS) by hand among a sample of Scottish cattle farmers, ultimately promoting the uptake of this behaviour through the design of an evidence-based behaviour change intervention.

The research process is summarised in Figure 2 below.

### The content analysis procedure: Stage 1. Coding of the farmers’ shared beliefs (gathered from the focus groups) using Interpretative Content Analysis

**Figure 3.**
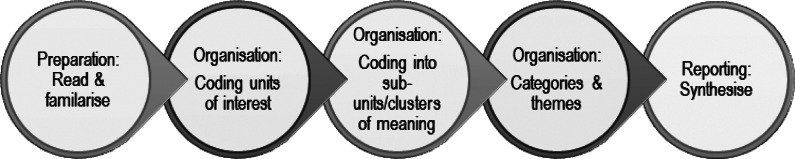
The five phases of the interpretative content analysis utilised by the authors for identifying the barriers and drivers to body condition scoring (BCS) among Scottish suckler cattle farmers.

Interpretative content analysis was adopted because it allows researchers to analyse what role(s) discourse plays in the everyday lives of the individuals, e.g. in terms of negotiations, decisions and meaning-making (Krippendorff 2004). The transcripts were deductively and inductively content analysed by LJ and the categories/themes were subsequently reviewed by ST. The content analysis followed the standardised approach of: (1) Preparation; (2) Organisation and (3) Reporting (see Figure 3) (Elo & Kyngäs 2008; Elo *et al.* 2014; Erlingsson & Brysiewicz 2017). LJ engaged in reflexivity throughout the coding process, recognising and embracing her own subjectivities in the analytical process. LJ kept a reflexivity journal documenting all her interpretative decisions. As the principal coder is a Scottish female, an academic psychologist, not from a farming background, and was not an animal welfare scientist, she recognised how her interpretations (i.e. what she saw/coded and how it was seen/coded) were influenced by her social and academic background, gender identity and world views. The full content analysis coding approach is provided in File B (see Supplementary material).

In the reporting and synthesising phase, the categories or themes were listed alongside their respective quotes and ST reviewed the list to ensure the quotes had been interpreted correctly and the farmers’ shared meanings or experiences were accurately reflected by the category/theme they had been coded to. A table of all the categories/themes is also provided in Table S1 in File B of the Supplementary material. LJ and ST then engaged in two rounds of discussions of each category/theme and their respective quotes to ensure there was agreement that the quotes had been correctly interpreted, coded, and assigned the correct category/theme and correct barrier/driver designations. One category/theme (i.e. *BCS by eye is easier than by hand*) was recoded, and the codes subsumed under a different category/theme. Consensus for the remaining categories/themes was reached in the second round of discussions.

### The BCW intervention mapping and design procedure: Stage 2. Mapping farmers’ beliefs onto the TDF domains and constructs

**Figure 4.**
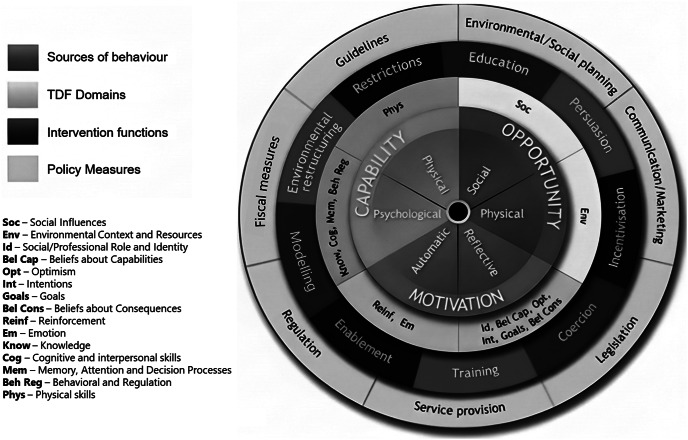
The Behaviour Change Wheel, reproduced with permission from Michie *et al.* (2014).

Mapping barriers and drivers of a target behaviour onto both the TDF (and the COM-B; Figure 4) is key to developing a data-driven behaviour change intervention (Michie *et al*. 2014). In line with previous research (e.g. Francis *et al*. 2009; Campbell *et al*. 2018), three academic psychologists (i.e. two independent academics and LJ) independently mapped the categories/themes to the TDF domains/constructs. The psychologists each had over twenty years’ experience researching and teaching in psychology theory (e.g. social psychology, social cognition, neuropsychology, psycholinguistics, emotion, child development, learning theory, psychometrics, and health psychology). This experience enabled nuanced distinction between overlapping TDF constructs and clear communication of the mapping rationales. The instructions and response template given to the reviewers can be found in File C of the Supplementary material.

After the reviewers mapped the barriers and drivers onto the TDF domains and constructs, they discussed issues of uncertainty or indecision in their mappings (e.g. where a reviewer felt that a TDF construct could be social norms or group norms). We should note that this stage was not designed to influence decisions but to identify if further consensus could be reached, to discuss some of the reasons for the final mappings and to share indecisions.

All domains and constructs reached agreement by at least two reviewers. Fleiss’s Kappa statistic was calculated to determine inter-rater reliability between the reviewer’s TDF mappings (Fleiss 1971). The data were analysed using IBM SPSS® Statistics for Windows, version 29 (IBM Corp 2023). Since the TDF domains and constructs directly inform which intervention functions and behaviour change techniques are selected, identifying agreement between the reviewers is an important stage in the intervention design. Inter-rater reliability (IRR) is therefore employed as another step in the systematic BCW mapping process.

### Stage 3. Linking the TDF domains and COM-B components (i.e. the COM-B diagnosis) onto the BCW intervention functions

In stage 3, LJ mapped the final TDF domains from Stage 2 onto their respective COM-B components to identify which broader behavioural influences need to change to perform the target behaviour (Michie *et al*. 2014). As the TDF maps directly onto the COM-B this can be done objectively, e.g. TDF: *Beliefs about Consequences* maps directly onto COM-B *Reflective Motivation.* Having the final ‘COM-B diagnosis’ we were then able to follow the relevant BCW step (5) (Michie *et al*. 2014), to link the behavioural diagnosis with intervention functions that have been identified as effective. Intervention functions refer to broad categories by which a specific intervention can change behaviour (e.g. see the red layer of the behaviour change wheel; Figure 4). As noted by Michie *et al*. (2014) the behavioural diagnosis that results from the COM-B and TDF analysis (Stage 2) is a key ‘staging post’ for designing an intervention.

### Stage 4. Linking BCW intervention functions to policy categories and applying APEASE criteria to both

In our fourth stage of the intervention design, we followed step 6 of the BCW guide (Michie *et al*. 2014), which is mapping intervention functions onto their respective policy categories. When the mapping identifies several potential options, researchers should apply the Affordability, Practicability, Effectiveness/cost-effectiveness, Acceptability, Side-effects/safety, and Equity (APEASE) criteria to select which specific intervention(s) to pursue (Michie *et al*. 2014). For full descriptions of the APEASE criteria please see Michie *et al*. (2014). The interventions and policy categories were therefore discussed amongst ST and KR. Both ST and KR are animal welfare scientists who have worked closely with our target audience for several years on the target behaviour. They were therefore ideally placed to assess the feasibility of the interventions and policy categories. Both researchers applied the APEASE criteria to the policy categories and intervention functions (e.g. is the intervention practical in the real world?) The policy categories and intervention functions identified as potentially feasible/effective, were then taken forward into stage 5 of our intervention design.

### Stage 5. Linking the COM-B, TDF and intervention function onto the Behaviour Change Techniques

In stage 5, the intervention functions and policy categories identified as most feasible in the preceding stage (4) were mapped onto the Behaviour Change Techniques (BCTs). The BCTs have been previously judged by an expert panel of judges as potentially effective for specific TDF domains and intervention functions (Michie *et al*. 2013; Cane *et al*. 2015). BCTs are described as “*an observable, replicable and irreducible component of an intervention designed to alter or redirect causal processes that regulate behaviour; that is, a technique is proposed to be an ‘active ingredient’*” (e.g. feedback, self-monitoring, reinforcement) (Michie *et al*. 2013; p 82). Given that both our intervention functions and TDF domains were identified through a rigorous review and analysis of the data we decided that first linking the BCTs to the TDF domains and then following up with a “checking” stage of linking the intervention functions with the BCTs would allow us to identify the most potentially effective BCT for our proposed intervention. Identifying the appropriate BCT also requires consideration of its appropriateness for the target audience, the context, and the intervention format. LJ and CN assigned the full BCW diagnosis (COM-B, TDF, intervention functions and policy categories) onto the appropriate BCTs as guided by the BCW steps (Michie *et al*. 2014).

Where there was disagreement between the BCTs, LJ and CN discussed these differences until a consensus was reached. CN also reviewed the proposed ‘real-world’ intervention against the BCTs identified to ensure that the intervention met the standardised BCT criteria (Michie *et al*. 2018).

As the BCTs have been designed to improve human health/well-being we adapted the terms so that they were aimed at improving animal health/well-being (e.g. BCT 5.1: Information about *animal* [added] health consequences). Each of the BCTs unique identifiers, as they are logged in the Behaviour Change Intervention Ontology (BCIO) (https://www.bciosearch.org), were also identified at this stage of the intervention mapping and design. An overview of the BCTs were given to ST and KR, and they were asked to also apply the APEASE criteria to these key BCTs.

### A summary of the research process

Given the number of steps involved in the intervention design process we have included Figure 2 to illustrate each of the steps taken in this current study, i.e. from initial data collection to intervention design.

## Results

### Stage 1. The content analysis findings and the identification of the barriers and drivers of BCS by hand

The content analysis identified 14 categories/themes which were identified as either a barrier or driver of BCS by hand, i.e. our target behaviour. Overall, we identified 11 barriers (e.g. *What is the actual point & uncertainty about how to body conditions core by hand*) and 3 drivers (e.g. *Seeing the benefits of body condition scoring by hand and we would like to learn*).

### Stage 2. Mapping farmers’ beliefs (categories/themes) onto the TDF domains and constructs

The reviewers’ mapping of the categories/themes onto the TDF domains and constructs revealed ‘moderate’ to ‘very good’ agreement. All domains and constructs had at least two of the three reviewers agree. There was moderate overall agreement between the reviewers in their mapping of beliefs to the TDF domains (Fleiss’s Kappa = 0.455; 95% CI: 0.305 to 0.605; *P* < 0.001). Individual kappa revealed good agreement for Environmental context and resources, (Κ = 0.717; 95% CI: 0.415 to 1.00; *P* < 0.001), and Beliefs about consequences, (Κ = 0.692; 95% CI: 0.252 to 0.857; *P* < 0.001). We also found moderate agreement both for Knowledge (Κ = 0.475; 95% CI: 0.475 to 0.777; *P* < 0.005) and for Behavioural regulation (Κ = 0.475; 95% CI: 0.475 to 0.777; *P* < 0.005). When assessing overall agreement between reviewers regarding TDF construct mapping, agreement was ‘fair’ (Κ = 0.333; 95% CI: 0.219 to 0.447; *P* < 0.001). Individual kappa revealed ‘moderate’ agreement for Person × Environment interaction (Κ = 0.546; 95% CI: 0.244 to 0.848; *P* < 0.001), Action planning (Κ = 0.475; 95% CI: 0.173 to 0.777; *P* < 0.005), Resources/Materials resources (Κ = 0.447; 95% CI: 0.145 to 0.750; *P* < 0.005), and Outcome expectancies (Κ = 0.417; 95% CI: 0.114 to 0.719; *P* < 0.01). Reviewers’ mappings, disagreements and agreements can be found in Table S2 in File D of the Supplementary material.

### Stage 3. Linking the TDF domains and COM-B components onto the BCW intervention functions

The COM-B diagnosis revealed Psychological capability, Physical opportunity, Automatic motivation and Reflective motivation as key influences of our target behaviour. An illustration of how the farmers’ beliefs, concerns and opinions mapped directly onto the COM-B components can also be found in Figure S2 in File E (see Supplementary material). In situations when there are several relevant COM-B components identified from the data, knowing which components to consider in terms of developing an intervention does require judgement on the part of the researchers involved and/or by drawing on past research (Michie *et al*. 2014). As there was no previous research in this area to help guide our decisions about which influences would be most relevant to target, we adopted the criterion of linking the TDF domains with good to moderate strength of agreement to their intervention functions. However, we should note that low or no agreement in the review process does not suggest that a factor is irrelevant in driving or impeding a desired behaviour. Thus, we did not automatically exclude those TDF domains with low inter-rater agreement, instead we also returned to the dataset and checked how often and how emphatically our participants expressed this opinion/belief to ensure we were not excluding a key influence of our target behaviour.

The TDF domains identified were Environmental context and resources (COM-B: Physical opportunity), Knowledge (COM-B: Psychological capability), Behavioural regulation (COM-B: Psychological capability), and Beliefs about consequences (COM-B: Reflective motivation). This meant that Automatic motivation was no longer included in our intervention design.

Guided by the BCW, we matched our key COM-B components to their respective Intervention functions (see Table 1). We also linked our TDF domains and constructs to the interventions, which allowed for an additional means of abstraction. The two Intervention functions that were not relevant for our overall intervention design were *Coercion* and *Incentivisation.*

**Table 1.** Linking the key COM-B components and the Theoretical Domains Framework’s domains/constructs (i.e. “the COM-B” diagnosis) to their respective Intervention Functions

BCW Mapping			Intervention Functions								
COM-B	TDFDomains	TDF Constructs	Education	Persuasion	Training	Restriction	Environmental Restructuring	Modelling	Enablement	Incentivisation	Coercion
Psychological Capability	Knowledge	Procedural Knowledge									
	Behavioural Regulation	Action Planning									
Physical Opportunity	Environmental Context & Resources	Resources/Material Resources Person x Environment Interaction									
Reflective Motivation	Belief about Consequences	Outcome Expectancies									

(adapted from Michie *et al*. 2014).

*Note:* COM-B refers to the Behaviour Change Wheel’s core framework. The ‘COM’ acronym represents the three components of behaviour: *Capability, Opportunity* and *Motivation.* The ‘B’ in COM-B refers to *Behaviour.*

The intervention functions that our COM-B /TDF diagnosis mapped onto are illustrated by the shaded cells in Table 1. The intervention functions identified were Education, Persuasion, Training, Restriction, Environmental restructuring, Modelling and Enablement.

### Stage 4. Linking BCW intervention functions to policy categories and applying the APEASE model

Table 2 illustrates how the intervention functions identified in Stage 3 map onto the recommended BCW policy categories.

**Table 2.** A matrix table showing the links between the intervention functions (identified from COM-B diagnosis) and the Behaviour Change Wheel’s policy categories (shaded cells)

Intervention Functions identified from our COM-B diagnosis	Policy Categories						
	Communication/Marketing	Guidelines	Fiscal Measures	Regulation	Legislation	Environmental/ Social Planning	Service Provision
Education							
Persuasion							
Training							
Restriction							
Environmental Restructuring							
Modelling							
Enablement							

(adapted from Michie *et al*. 2014).

*Note:* COM-B refers to the Behaviour Change Wheel’s core framework. The ‘COM’ acronym represents the three components of behaviour: *Capability, Opportunity and Motivation*. The ‘B’ in COM-B refers to *Behaviour*.

The APEASE criteria were subsequently applied to each of the identified policy categories, and intervention functions, as illustrated in Table 3.

**Table 3.** Application of the APEASE criteria to the identified policy categories

	A	P	E	A	S	E
	Acceptability	Practicability	Effectiveness	Affordability	Spill-over	Equity
Intervention						
Education	high	high	high/medium	high/medium	+ve	none
Persuasion	high	medium	medium	high/medium	+ve	none
Training	high	high/medium	high/medium	high/medium	+ve	none
Restriction	low	low	medium/low	low	-ve	<
Environmental Restructuring	medium	medium	medium	medium	+ve	none
Modelling	high	high	high/medium	high/medium	+ve	none
Enablement	high	high	high/medium	high/medium	+ve	none
Policy						
Communication/Marketing	high	high	medium	high	+ve	none
Guidelines	high	high	high/medium	high	+ve	none
Service Provision	high	medium	high/medium	medium/low	+ve	<
Fiscal	low	low	high	low	+ve	none/<
Regulation	low	low	medium/low	low	-ve	<
Legislation	low	low	medium/low	medium	-ve	<
Environmental social planning	medium/low	low	medium	low	+ve	<

(adapted from West *et al.* 2020)

Key: -ve = potentially negative; +ve = potentially positive; < = potential decrease in equity; > potential increase in equity. Note, “none” refers to the intervention function or policy category as having an unlikely effect on equity i.e. decreasing or increasing the differences between advantaged and disadvantaged farmers.

*Note:* The APEASE criteria acronym are Acceptability, Practicability, Effectiveness, Affordability, Side or Spillover effects, and Equity.

The shaded boxes in column one of Table 3, indicate which intervention functions and policy categories sufficiently met the APEASE criteria. These were taken forward to stage 5 of the intervention design.

### Stage 5. Linking the intervention functions and TDF domains to the BCTs

In this fifth stage of the intervention design, we mapped the intervention functions and TDF domains onto their respective BCTs as guided by the BCW. Each stage of the BCW mapping is illustrated in Table 4. Only those BCTs we deemed most feasible for our target behaviour and target audience are listed in the table. However, a full list of the BCTs that have been previously matched to these particular TDF domains and intervention functions can be found in the Supplementary material (see Table S3 in File F).

**Table 4.** Summary of the complete Behaviour Change Wheel intervention mapping: linking COM-B components, Theoretical Domains Framework Domains/Constructs, Intervention Functions, Policy Categories, and Behaviour Change Techniques for promoting body condition scoring in Scottish suckler cattle farmers

COM-B	TDF:Domains	TDF: Constructs	Intervention Function	Policy Category	BCT
Psychological Capability	Knowledge 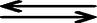	Procedural Knowledge 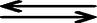	Education 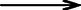	Communication/ Marketing, Guidelines & Service Provision 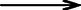	**(2.2)** Feedback on behaviour (BCIO:050661) **(5.1)** Information about animal health consequences (BCIO:007063) **(5.3)** Information about social & environmental consequences (BCIO:007064 & BCIO:007176) 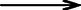
	Behavioural Regulation 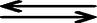	Action Planning 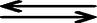	Training, Modelling & Enablement 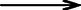	Guidelines & Service Provision 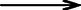	**(6.1)** Demonstration of the behaviour (BCIO:007055) **(4.1)** Instruction on how to perform a behaviour (BCIO:007058) **(2.3)** Self-monitoring of behaviour (BCIO:007024) **(1.4)** Action Planning (BCIO:007010) 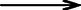
Reflective Motivation	Beliefs about Consequences 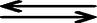	Outcome Expectancies 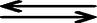	Education, Persuasion & Modelling 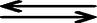	Communication Marketing, Guidelines & Service Provision 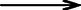	**(5.3)** Information about social & environmental consequences (BCIO:007064 & BCIO:007176) **(9.1)** Credible source (BCIO:007075) **(5.1)** Information about animal health consequences (BCIO:007063) 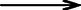
Physical Opportunity	Environmental Context & Resources 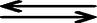	Resources/ Material Resources	Training, Environmental Restructuring & Enablement 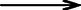	Guidelines & Service Provision 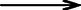	**(4.1)** Instruction on how to perform a behaviour (BCIO:007058) **(2.2)** Feedback on the behaviour (BCIO:007022) **(8.1)** Behavioural practice/Rehearsal (BCIO:007094)
		Person x Environment 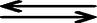			**(7.1**) Prompts/cues (BCIO:007080) 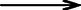

*Note:* the BCT numbers refer to how they are listed in the BCT Taxonomy alongside their unique BCIO identifiers. COM-B refers to the Behaviour Change Wheel’s core framework. The ‘COM’ acronym represents the three components of behaviour: *Capability, Opportunity and Motivation*. The ‘B’ in COM-B refers to *Behaviour*.

We should note that the arrows in Table 4 denote how each intervention design stage – as guided by the BCW – maps onto each other, e.g. Psychological capability maps onto TDF domain Knowledge; the specific TDF construct for Knowledge is Procedural knowledge. Based on this COM-B diagnosis, we selected the intervention function of Education, delivered through the policy categories of Communication/Marketing, Guidelines, and/or Service provision. In the final column are the BCTs which are the active ingredients of our identified interventions. The bidirectional arrows (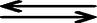) between COM-B and TDF indicate that these frameworks have fixed theoretical relationships. For example, Knowledge (TDF) always maps to Psychological capability (COM-B) and *vice versa.* This means that whether we begin our analysis at the COM-B or TDF level, the theoretical diagnosis remains consistent. In contrast, unidirectional arrows (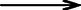) show our forward design process from theoretical diagnosis to intervention selection. As illustrated in the table there is also significant overlap between the intervention functions, policy categories and BCTs identified, which will allow for more a consolidated intervention design overall.

### The final stage of our intervention design process: Our proposed intervention

Drawing on the BCTs that we identified from our intervention design in Stages 1–5, we designed a potential intervention, which we aim to implement and evaluate. In this final step of our intervention design, we identified which BCTs will best serve our intervention functions. We also know from previous stages (i.e. Stage 4) which mode of delivery is most appropriate to implement our intervention (i.e. Guidelines, Service provision and Communication and Marketing). As noted by Michie *et al*. (2018) it is possible to include more than one BCT in a single intervention. A more detailed account of the proposed intervention alongside BCT criteria, notes and a definitions table can be found in File F (see Supplementary material).

In summary, our proposed intervention – as signposted by our findings – will aim to include written guidelines outlining the animal health consequences, the economic, social and environmental consequences of BCS by hand and provide action plans on how to, when to, and how often to BCS by hand. Training in, and demonstration of, the target behaviour are also key intervention functions to consider in our final intervention plan. We will ask farmers to monitor their BCS activities and provide them with meaningful feedback. We will also aim to engage farmers in feedback discussions and peer-support groups/networks to help us identify what does and does not work in terms of our interventions and to ensure farmers become co-designers in future intervention development.

## Discussion

Our work shows that the Behaviour Change Wheel (Michie *et al*. 2014) provides an ideal framework for developing an evidence-based intervention, designed to elicit positive behaviour change. As discussed, there is an increasing body of evidence regarding why farmers do (or do not) engage in certain behaviours (Balzani & Hanlon 2020), or foster specific attitudes, perceptions or beliefs and how they make decisions (Austin *et al.* 2005; Bock & Van Huik 2007; Kielland *et al.* 2010; Kauppinen *et al*. 2013; Balzani & Hanlon 2020). More recently, research has examined how these influences on behaviour can be mapped onto human behaviour change models, such as the BCW, to create interventions that could lead to positive animal welfare outcomes (McDonald *et al.* 2018; Furtardo *et al.* 2022; Clark *et al.* 2024). Despite researchers recommending that systematically designed behaviour change interventions are necessary to elicit lasting positive welfare outcomes (see Carroll & Groarke 2019; Carroll *et al.* 2021), few studies go beyond the COM-B diagnosis stage (McDonald *et al.* 2018), with some stopping short of identifying the theoretical underpinning of their intervention (Glanville *et al.* 2020). What also remains under-researched is the implementation and testing of the interventions (Glanville *et al.* 2020). This is not a criticism of these studies, as we recognise a full intervention design process is time consuming and intervention implementation and testing even more so. In this study, our aim was to go beyond the COM-B diagnosis to create a potential intervention that *could* subsequently be implemented and assessed. We were able to do this because we were fortunate enough to have the time, and an interdisciplinary team of researchers and reviewers to contribute to the process of designing a full intervention.

We therefore drew upon farmers’ self-reported behavioural influences, including beliefs, experiences, attitudes and opinions, to design an intervention to promote BCS of suckler cattle by hand. We utilised all components of the Behaviour Change Wheel framework (Michie *et al*. 2014), including the COM-B model, the Theoretical Domains Framework, Intervention functions, Policy categories and the Behaviour Change Technique Taxonomy/Ontology (Michie *et al*. 2014).

The TDF served as a particularly useful framework in our intervention design. Looking at the data through the TDF lens, allowed for an additional layer of abstraction, checking and validation. For example, Psychological capability which mapped onto Knowledge, which then mapped onto *Procedural knowledge* forced us to think about what type of knowledge was most relevant. For example, demonstrating to farmers exactly ‘how’ to BCS by hand (i.e. procedural knowledge) is more likely to be effective at enhancing psychological capability than simply providing written guidelines that increases knowledge of body condition scoring in general. Past research has also shown that providing ‘how-to’ guides increases the recipient’s confidence in their perceived ability to engage in the target behaviour and is therefore more successful than only providing information on ‘why’ to engage in the behaviour (Pope *et al*. 2018). Psychological capability also distilled onto the *Behavioural regulation* domain, which according to the BCW is best served by training, modelling, and/or enablement. Further, the TDF construct for *Behavioural regulation* identified by our reviewers was *Action planning*, which mapped onto the belief/theme: “We would like to learn”. Action planning has also been identified as important in the implementation phase of behaviour change (Pelletier & Sharp 2008; Pope *et al*. 2021). In this phase of change, the individual has already established intentions to change their behaviour but may lack the confidence to act (Pope *et al*. 2018). Thus, to turn an individual’s intentions (e.g. we would like to learn) into behaviours, they must be supported in developing the confidence to act (Rothman *et al.* 2006; Pelletier & Sharp 2008; Pope *et al.* 2018). Showing farmers how to BCS by hand through training and modelling may thus be effective in eliciting change but providing an action plan will likely be more effective at maintaining the behaviour change. Action plans, for example, provide additional information such as context, frequency, duration and/or intensity of the target behaviour (Michie *et al.* 2014). The medium for improving psychological capability through increased or improved knowledge is clearly important such that specific approaches to farmer education will determine the success of these education-based interventions. In summary, the more capability is increased through practice/rehearsal of condition scoring by hand, the more one might feel motivated to enact the behaviour, forming a positive feedback loop between behaviour, capability and motivation (West & Michie 2020). The motivation to engage in the desired behaviour (body condition scoring by hand in addition to eye) must also exceed that of the motivation to engage in a competing behaviour (only assessing condition by eye, or not body condition scoring at all) (Michie *et al.* 2014). As such, it is important for us to address motivation as an integral influence of behaviour. Indeed, *reflective motivation* was the barrier that we observed most frequently overall and forms the central focus of our intervention design. Reflective motivation consistently distilled onto Belief about Consequences (TDF domain), and Outcome expectancies (TDF construct). These are the subjective perceptions of the outcomes and consequences of a behaviour in a given setting (Cane *et al.* 2015). This TDF domain also maps onto the Education, Persuasion and Modelling intervention functions. The categories/themes to which this TDF was identified were “What is the actual point?”; “No point condition scoring pre-calving”; “No point BCS as putting them into groups causes more problems” and “Weight loss is just the natural consequence of a mother feeding her calf/calves”. The central organising concept for all these categories/themes was a belief or perception that there is little or no point routinely BCS by hand. As such, this points to low motivation to change. We believe this is an integral barrier to tackle in our intervention because without motivation, no amount of increasing capability and improving opportunity will achieve our target behaviour. We should however also note that reduced motivation often stemmed from the beliefs about the problems that manifest from grouping cows. In this instance, to resolve the problem of low motivation would also mean helping farmers to resolve their cow grouping problem via environmental restructuring or enablement, for example. We later return to this problem in our discussion of physical opportunity.

Persuasion, which was one of the intervention functions linked to reflective motivation, is defined as changing the way people feel about a behaviour by generating cognitive dissonance (Michie *et al*. 2014). To note, cognitive dissonance is the unpleasant psychological state that results from inconsistency between two or more elements in a cognitive system (Festinger 1962). To create dissonance in farmers who simply “don’t see the point", we would need to provide them with a new “there is a point” understanding of BCS. Michie *et al.* (2014) also note that an intervention can have more than one function, so by educating a farmer on how to BCS by hand, we may also persuade them that there is a point to BCS by hand.

Finally, Physical opportunity was mapped onto the TDF domain Environmental context and resources. This domain pertains to conditions in a situation or an environment that facilitate or hinder the occurrence of a behaviour (Cane *et al.* 2012). The TDF constructs for this domain were also identified as Resources/Material resources and Person × Environment interaction. This domain was then mapped onto Training, Environmental Restructuring and Enablement intervention functions. The identification of the physical opportunity component came from the farmers’ recognition that they did not have the resources to split livestock into groups after BCS. In this context, training and restriction would not be appropriate interventions. Instead, one would need to put environmental restructuring in place, specifically providing farms with pens for split grouping. After the APEASE criteria were applied, this intervention was identified as too costly to be feasible. Although one suggestion from our animal welfare experts was that farmers could split their existing pens with surplus farm gates. This is perhaps something that could be explored in the future. Another physical opportunity barrier was also identified by those who stated it was not possible to BCS when their cattle are in the field. Although the reviewers’ interpretation of this theme led to it being mapped as a physical opportunity barrier, the animal welfare researchers later pointed out that body condition scoring in a field is not necessary as most cows will be spring calvers and so will spend most of their pregnancy indoors. Making it clear to farmers when to BCS therefore appears pertinent. This possible misinterpretation also suggests this theme/category is not a physical opportunity barrier but more a barrier of psychological capability, specifically knowledge.

As previously noted, another intervention function that maps on to Environmental context and Resources domain is enablement, defined as providing support to improve ability to change, in ways not covered by other intervention functions (Michie *et al*. 2014). In this sense, enablement might also involve behavioural support for farmers, such as encouragement or support from other farmers, vets, or farm consultants. Support can also come from those individuals close to the farmer such as friends and family. The role vets play in influencing BCS by hand does warrant further examination. Although the participants were asked about whether vets encourage BCS by hand, very few participants responded to this question directly, instead the participants made jokes about the cost of vets.

The policy categories that we also identified to deliver our intervention were Guidelines, Communication and Marketing and Service provision. Guidelines are typically characterised as the development and dissemination of documents that make evidence-based recommendations for action for specific situations. Guidelines are also identified as most appropriate when there is a need to educate people about what needs to be done and why. Guidelines are also most effective when there is little or no resistance. The use of case studies to model good practice are also recommended (West & Gould 2022). Communication and marketing are typically characterised as mass media campaigns, digital marketing campaigns, and correspondence. Communication and marketing are most relevant when there is a need to educate people about what to do or why change is important, or to persuade them of its importance and to trigger action (West & Gould 2022). Service provision is the provision of services, materials and/or social resources. This policy category is most relevant when we wish to improve the individual’s ability to change their behaviour (West & Gould 2022). Our proposed intervention will therefore utilise all three of these policy categories.

Our aim in terms of implementing an intervention would therefore be to communicate the importance of BCS by hand ‘messages’ through farming press, knowledge exchange communications and newsletters. Signposted by our BCT mappings, our guidelines would also contain detail on the cattle health outcomes of engaging in BCS by hand (BCT 5.1 Information about ‘animal’ health consequences) and how BCS by hand can reduce feed waste (BCT 5.3 Information about social and environmental consequences). In our guidelines we would also aim to include endorsements/testimonials from credible sources (BCT 9.1 Credible source) and detailed planning of how to carry out BCS by hand, outlining clearly the context, frequency, and duration required (BCT 1.4 Action planning). In terms of service provision, our aim would be to run in-person and/or online workshops and farmer events organised by animal welfare scientists to demonstrate effective, efficient and accurate BCS by hand (BCT 6.1 Demonstration of the behaviour). Farmers should also be provided with the opportunity to practice (BCT 8.1. Behavioural practice/rehearsal). The effectiveness of this intervention would be measured through a case study group.

### Study limitations

One clear limitation in our study is that the data were drawn from a small sample of farmers and are therefore not representative of all suckler cattle farmers in Scotland. Although we recognise not having a representative sample is a limitation, we are confident that we uncovered the principal reasons most farmers do not BCS by hand. For example, the barrier of reflective motivation was so consistently observed across all focus groups that we suspect this is a belief held by most farmers who do not BCS by hand. Further, as this COM-B component (reflective motivation) underpins several of our proposed interventions and BCTs, we are confident our intervention should prove effective with other suckler cattle farmers.

We also recognise that 15 participants are at the upper end of what is typical for focus groups and may have resulted in not everyone having the opportunity to speak (Hennink *et al.* 2019). However, as these focus groups took place at farmer events, larger group gatherings tend to be the norm and have the capacity to foster friendly discussions. The participants in each focus group were also based in the same region in Scotland, and so most of the participants knew each other. As the farmers had also participated in other group sessions and had a lunch/refreshment prior to our focus group discussions, we were confident that all the participants felt comfortable to speak as part of the group. As aforementioned, the moderator also engaged in techniques to encourage whole group discussion and enable the quieter voices to be heard.

Another limitation is that farmers who attend workshops are potentially already engaged in making changes and thus provided us with a relatively biased sample. However, given the number of barriers we observed, we are confident that our groups were not necessarily all primed for change, e.g. in the contemplation, preparation or action stages of behaviour change (see Prochaska & DiClemente 1986).

Another possible limitation is that our focus group discussions focused too much on the barriers to body condition scoring by hand, rather than drivers. As noted by Michie *et al.* (2014), it is important to identify both barriers and drivers when developing an intervention. Although we did ask farmers about what they see as the potential benefits, our questions primarily focused on the barriers. Future studies should engage farmers in discussions around what they also see as the benefits, perhaps looking at more participatory action research (PAR: Gouttenoire *et al.* 2013; Brown 2022) to identify solutions to the problem.

Although information power (Malterud *et al.* 2016) was not part of our methodological plans; a retrospective assessment of our focus groups suggests adequate to moderate information power across some of the key information power dimensions: (1) relatively narrow research aims/focus (identify barriers/drivers of body condition scoring by hand and design an HBC intervention); (2) sample specificity (Scottish suckler cattle farmers); (3) reliable theoretical grounding (COM-B & Theoretical domains framework); and (4) systematic analysis of the focus group discourse (Interpretative content analysis). Future research might consider looking at information power to assess the quality and the relevance of the data, particularly when using interviews and focus groups to identify behavioural influences.

### Animal welfare implications

BCS is recommended for monitoring fat deposits, requiring the farmer to get their hands on to gain an accurate assessment of condition (Lowman *et al*. 1976). As only 4% of UK beef farmers use the hands-on assessment (SAC Commercial 2013) it is likely that some cows experience unacceptable body condition. The welfare implications of dropping below an acceptable body condition score are clear with lean cows experiencing more calving difficulties which is a known risk factor for poor calf vigour, calf immune development and health outcomes (Barrier *et al*. 2012; Diskin & Kenny 2016; Fordyce *et al*. 2021). Encouraging farmers to engage in a practice that could increase the number of cows identified as too lean or overweight will ultimately lead to more positive welfare outcomes for both the cow and her offspring.

## Conclusion

Given what we discovered in terms of the range and nature of the barriers and drivers of our target behaviour, we suggest that applications of social and behavioural science theory should go beyond the more individual-level approaches to understanding human behaviour in animal caretaking contexts.

Currently, there is a wealth of information about *what* animal caretakers do and do not do to improve the health and welfare of animals. However, much less is known about *why* people engage in positive or negative behaviours and even less is known about *how* we use this information to elicit positive behaviour change. The aim of future research in this field should be to identify how we can elicit and support best practice behaviours. We believe that psychology theory and the BCW can afford animal welfare research with the most accurate ways to understand the behaviours of animal caretakers and to effectively identify ways to encourage and support positive behaviours that persist over time. We therefore recommend an interdisciplinary approach, involving both animal welfare scientists, animal welfare practitioners and psychologists. Thus, although the BCW provides researchers with a step-by-step guide on how to design and implement an intervention, psychologists have an in-depth understanding of the foundational theories that underpin the TDF domains and constructs. An understanding of psychological theory and human behaviour served the psychologists well during the mapping of the categories/themes onto the TDF domains and constructs in the current study. Psychologists can also be valuable in the assessment of an intervention, specifically being able to identify the underlying processes through which a BCT may have elicited change (i.e. the mechanism of action).

The animal welfare scientists’ understanding of the target audience and target behaviour also proved invaluable in terms of interpreting and unpacking the farmers’ beliefs, opinions and experiences. Knowing which intervention would work in the ‘real world’ was also a valuable insight provided by our animal welfare scientists.

In conclusion, we believe bridging the gaps that exist between evidence and practice can be achieved through an interdisciplinary team of psychologists and animal welfare scientists. We propose both fields are well-positioned to leverage their complementary expertise to significantly improve animal welfare. We also recognise the value of carrying out rigorous COM-B and TDF analyses/mapping to understand the key barriers of best animal welfare practices. However, we would encourage researchers to build on this foundational work to design, implement, and assess interventions in real-world settings; essentially bridging the gap between ‘COM-B diagnosis’ and demonstrable improvements in animal welfare.

## Supporting information

10.1017/awf.2026.10072.sm001 Jessiman et al. supplementary material Jessiman et al. supplementary material

## Supplementary material

The supplementary material for this article can be found at http://doi.org/10.1017/awf.2026.10072.
