# Supplementary material for: Bridging the gap between human behaviour and animal welfare: A study on human behaviour change and body condition scoring of suckler cows
Source: Anim Welf. 2026 Mar 3;35:e17. doi: 10.1017/awf.2026.10072 (PMC12963783; doi:10.1017/awf.2026.10072)
Supplement: Jessiman et al. supplementary material [file S0962728626100724sup001.docx]

**File A.**

**The Farmer Focus Group Interview Schedule & the Full Farmer Questionnaire:**

**Focus-Group Interview schedule:**

- *Can we begin with just seeing how many of you currently body condition score your cattle?*
- *Follow on: how many of you do get your hands on to body condition score?*
- *Prompt: can you tell me why you get your hands on?*
- *Prompt: can you tell me why you don’t get your hands on?*
- *What about recording scores, do any of you formally record your body condition scores?*
- *Prompt: can you tell me why you record your scores/why you don’t record your scores?*
- *Does anyone speak to their vet about the condition of your cows?*
- *Prompt: can you tell us a little more about that; do they bring it up; do you bring it up?*
- *Does anyone speak to anyone else e.g. a nutritionist about condition? (all followed by prompts: why, why not?)*
- *Has anyone ever had any issues such as cows being over or underweight pre-calving?*
- *Prompt: Could you please elaborate?*
- *Could we now discuss what sorts of things people do when they find they have an underweight/overweight cow?*
- *Prompt: Could you please elaborate?*
- Is there anything we have not discussed that anyone thinks is relevant and would like to discuss now?

**Farmer Questionnaire:**

**
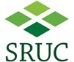
**

# Title: Perceived Benefits of Body Condition Scoring Suckler Cows & Barriers to its Adoption

The following questionnaire is designed to identify your current practices, beliefs and understanding about **body condition scoring**. As explained in your participant information sheet, all the information that we collect from you today will be anonymous and will remain completely confidential. If there are any questions you do not wish to answer, please simply leave blank and move on to the next question. Please note, some questions may not apply to you so please simply move on to the next questions as directed.

Demographic & Background Information

1. Age (years):

Farm Background

2. How many cows do you have in your herd? __________________________________

3. What time of year do your cows calve? Please tick the box(es) that applies/apply to you:

**Spring**

**Autumn**

___________________________________________________________________

Body Condition Scoring

4. Please tell us which body conditioning scoring method you use, when you use this method, and how frequently you use this scoring method below. Please tick the box(es) that applies/apply to you:

**I do not use body conditioning scoring**  - please skip to questions #9-11

**At housing or weaning, by hand**

| Not routinely |  |
| --- | --- |
| Only when I have time |  |
| Only when there is a problem with a cow |  |
| Never |  |

**At housing or weaning, by eye**

| Routinely |  |
| --- | --- |
| Not routinely |  |
| Only when I have time |  |
| Only when there is a problem with a cow |  |
| Never |  |

**At late pregnancy or pre-weaning, by hand**

| Not routinely |  |
| --- | --- |
| Only when I have time |  |
| Only when there is a problem with a cow |  |
| Never |  |

**At late pregnancy or pre-weaning, by eye**

| Routinely |  |
| --- | --- |
| Not routinely |  |
| Only when I have time |  |
| Only when there is a problem with a cow |  |
| Never |  |

5. Do you use a formal body conditioning scale? Please tick:

**No, I do not use a formal body conditioning scale**

**Yes**

**If yes, please specify how many categories you use in your scale (e.g., 5-point scale): _______________________________________________________**

6. Why do you use body conditioning scoring? Please tick all the box(es) that applies/apply to you:

**Guide feeding of the whole herd ☐**

**Tailor feed to individual animal needs ☐**

**Allow formation of groups based on body condition ☐**

**Other ☐ (please explain/specify)**

**___________________________________________________________________**

7. How do you record the cow’s condition score? Please tick all the box(es) that applies/apply:

**A handwritten recording ☐**

**An electronic recording ☐**

**I do not record the scores ☐**

8. Over the winter how are your cows grouped? Please tick all the box(es) that applies/apply to you?

| Only have one group |  |
| --- | --- |
| Group based on body condition |  |
| Group based on calving status |  |
| Group based on breed |  |
| No system for grouping |  |
| Other |  |

**If you stated “Other,” please explain/specify**

**___________________________________________________________________**

9. In the following table you will find a list of statements. Please read each of the statements carefully and then respond in terms of your level of agreement, disagreement, or neutrality about that statement.

|  | **Strongly Agree** | **Agree** | **Neither Agree nor Disagree** | **Disagree** | **Strongly Disagree** |
| --- | --- | --- | --- | --- | --- |
| **Body condition scoring is too complex to understand.** |  |  |  |  |  |
| **Most farmers I know, do not use a formal body conditioning scale.** |  |  |  |  |  |
| **Body condition scoring is too time consuming.** |  |  |  |  |  |
| **Body condition scoring is good for herd management.** |  |  |  |  |  |
| **I would benefit from more practice or experience body condition scoring.** |  |  |  |  |  |
| **Body condition scoring does little to improve cow welfare.** |  |  |  |  |  |
| **My vet has never mentioned the body condition of my cows.** |  |  |  |  |  |
| **I am aware of industry recommendations for appropriate body condition scores, e.g., target score before calving.** |  |  |  |  |  |
| **Body condition scoring improves how well calving goes.** |  |  |  |  |  |
| **I know exactly how to body condition score and exactly which parts of the body to focus on.** |  |  |  |  |  |
| **Body condition scoring can lead to a financial benefit for my farm business.** |  |  |  |  |  |
| **I have little opportunity to change the feed that I give to the cows, so body condition scoring is of no help to me.** |  |  |  |  |  |
| **Body condition scoring by eye is just as accurate as scoring using the hands.** |  |  |  |  |  |

We are very interested in learning from farmers’ lived experiences. Please take a few moments to elaborate on your experiences of, and beliefs about, body conditioning scoring. Your time and consideration are very much appreciated.

10. Please write below any reasons you feel body condition scoring can be challenging (or is not necessary) if these have not been covered previously in the questionnaire.

___________________________________________________________________________________________________________________________________________________________________________________________________________________________________________________________________________________________________________________________________________________________________________________________________________________________________________________________________________________________________________________________________________________________________________________________________________________________

11. Please write below any other benefits you feel that body condition scoring can bring if these have not been covered previously in the questionnaire.

________________________________________________________________________________________________________________________________________________________________________________________________________________________________________________________________________________________________________________________________________________________________________________________________________________________________________________________________________________________________________________________________________________________

Thank you for taking the time to complete this questionnaire.

**File B.**

**The full content analysis coding approach**

**Background:**

- We adopted Interpretative Content Analysis (Ahuvia, 2001).
- The interpretive approach allows the coder to consider context and explore shared beliefs and patterns of meaning that are not signposted by predetermined research questions or only guided by the surface level meaning of discourse (i.e. manifest coding).
- Manifest and latent coding were both used in our analysis as part of the iterative process (i.e. the type of content coded).
- We also adopted deductive and inductive coding (i.e. the method of code generation).
- Deductive content analysis was guided by our research questions i.e. what are the barriers and drivers of body condition scoring cattle by hand?
- With interpretive content analysis, inter-rater reliability is not sought, particularly because this approach is misaligned with the post-positivist epistemology of qualitative research. Instead, transparency and quality control of coding is managed by readers, reviewers, and co-authors (Ahuvia, 2001).
- In adopting this approach, we worked also within “hermeneutic circles,” acknowledging the influence of our own social and cultural backgrounds in the coding and interpretations of the data (Krippendorff, 2004). As the principal coder is a Scottish female, a psychologist, not from a farming background, and was not an animal welfare scientist, she recognised how her interpretations were influenced by her social background, gender, and world views.
- Interpretive content analysis does not falsely claim objectivity; recognising this is not possible even with more traditional content analysis approaches. Instead, as with more recent qualitative approaches, researchers embrace subjectivity as an important and recognised part of the analysis and interpretation.
- Public justifiability is used in interpretative content analysis whereby the researchers explain their approach, acknowledge their subjectivities and reveal to the reader(s) their coding to show that their interpretations are in the very least plausible even in the face of alternative interpretations (Ahuvia, 2001).

**The Interpretive Content Analysis Process:**

The defined recording units in our content analysis were full sentences, paragraphs, and phrases extracted from the full transcripts from all six focus groups and from the answers to the questionnaire’s open-ended questions e.g., ‘please tell us any reasons you feel body condition scoring can be challenging or is not necessary’ and ‘please tell us what you see as the benefits of body condition scoring’.

- **Preparation phase:**
- The coder immersed herself in the data by reading each of the transcripts several times, familiarising herself with the text and considering and reflecting upon what was stated/shared/voiced in the transcripts.
- Whole transcript/co-text coding was adopted to allow for a more accurate interpretation of meaning. It also allowed the coder to consider context.
- **Organisation phase:**
- The analysis was non-linear and iterative in approach.
- During coding, NVivo for windows version 1.7 pro (Lumivero, 2023) was used to manage the data.
- We began with deductive coding of the data for points directly related to our research questions. Research questions: 1. What are the barriers and drivers of body condition scoring by hand? 2. What are farmers’ perceptions and beliefs around the conditioning of their cows?
- We therefore examined the text for expressed beliefs, opinions, and perceptions around our research questions.
- The coder first engaged in manifest coding: coding from the explicit/surface level meaning of the text i.e. what the participant explicitly stated.
- We also adopted an inductive coding approach looking for points of interest that transcended our research focus of barriers and drivers of body condition scoring by hand.
- The coder made memos and kept note of the relevant codes and categories/themes as well as highlighted sections of the text that provided more context, which assisted in the interpretation of meaning.
- Forming of sub-categories from the data by grouping together codes that were related to each other by content or context.
- The coder looked for links between the sub-categories to form superordinate categories.
- In the final stage of abstraction, the researcher also engaged in latent coding to identify some themes. Latent coding is when one "ferrets out a text’s subtler meanings” (Ahuvia, 2001, p.141).
- **Data was coded to category and theme levels:**
- a category reflects the *who, what, when, or where*
- a theme reflects the *why, how, in what way, or by what means* (Elo et al., 2014; Elo & Kyngäs, 2008; Erlingsson & Brysiewicz, 2017).
- A coding principle that we adhered to was that a category or theme could only be formed if it had a minimum of two codes. Although some may argue that two codes are not sufficiently rigorous, we reasoned that for at least two farmers to note something as relevant to bring it into the focus group discussions, should qualify it as relevant and meaningful at least in this stage of our analysis.
- Our analysis was exhaustive such that all data were coded.
- Where a code could not be assigned to a category or theme these codes were labelled as “other” (Krippendorff, 2004)
- Due to the lack of relevance that “other data” had to our research questions these data were not included in the intervention mapping. In the most part, “other” data included off-topic discussions.
- All categories/themes from the content analysis were assigned titles or labels that the researcher felt best reflected the farmer’s internal constructs such as their beliefs, perceptions, or attitudes towards body condition scoring by hand and reflected the farmers’ shared beliefs, attitudes, and concerns. The researcher primarily engaged in manifest coding i.e. coding from the surface level meaning of the text thus identifying categories based on what someone explicitly said.
- The initial coding, categories and themes were listed alongside their respective quotes and reviewed by one of the co-authors (as illustrated in the following table).
- Consensus meetings took place between the coder and the co-author to discuss any disagreements or ambiguities. Two rounds of discussions took place before finalising the categories and themes.

The following supplementary table illustrates the initial coding of categories, and the final coding of superordinate categories and themes. The table also illustrates whether these categories/themes are barriers or drivers of body condition scoring by hand.

**Supplementary Table S1. Example Quotes, initial coding, final categories, and themes from the interpretive content analysis of the barriers and drivers of body condition scoring by hand.**

| **Example Quotes** | **Initial Coding(s)** | **Initial Categories/Themes** | **Categories/Themes after discussions** | **Barriers or Drivers of Body Condition Scoring** |
| --- | --- | --- | --- | --- |
| *“What's the fascination with getting at the side of the cow and poking about?”*  *“what’s the advantage? when you look at it through, hands off”.*  *“Putting my hands on I don’t know what difference that is going to make. That’s all I can say”.* | Why do we need to do it?  What's the actual point of doing it by hand?  We just do not see point of body condition scoring by hand. | What’s the point? | What is the actual point? | Barrier |
| *“So, are you no better to actually if you’ve got these lean cows are you no actually better to yes contain them and keep them going through with minerals and stuff like that and then once they calve then start adding supplement feeding onto them to then start to build them up for going out to grass…”* | No point Condition Scoring Cattle Pre-Calving  It is more about feeding after calving, not BCS before calving.  Wait until after. | No point pre-calving time | No point Condition Scoring pre-calving | Barrier |
| *“Doing it by hand is more difficult because not sure what the right condition should feel like where with eye, I am more practiced/knowledgeable with that method”.*  *“When you say regularly, do you mean, how many times do you think?”*  *“What is the ideal?”*  *“Doing it by hand is more difficult because not sure what the right condition should feel like. Where with eye we know how to do that”* | Uncertainty or Lack of Skill/Knowledge  Uncertainty about how to body condition score by hand. | Not sure how to do it | Uncertainty about how to score by hand | Barrier |
| *“I mean I have been dealing with cattle all my life and I think I know when, whether it’s, good or bad”.*  *“And you know your cows. So, you’re no needing to be putting them through a crush handling them to know whether they’re too fat or too thin”.*  *“40 years’ experience, I know my cows”* | By Eye is OK because we “Know our Cows”  I have been doing this for a long time.  I know when my cows are fat or thin | I know my cows | Not necessary as we know our cows | Barrier |
| *“Well, there were some cows last year that I thought were looking on the lean side. I put them through the crush. I condition scored them and they were all above the condition score I thought they were at. I was miles out”.*  *“And so, I was thinking they were pretty lean but actually, but actually they were exact same weight as where they were that should tell you and asking week has been around should tell you the lessons, we've gotten ours are”.*  *“Yeah yeah, you think she’s quite thin then you put your hand on and she’s not”* | Confirmed belief that by eye is OK  They were fatter than I thought  Not necessary as even fatter when scored just by eye | By eye is enough | They are usually fatter than you perceive by eye | Barrier |
| *“You’re right though the cows tend to be a condition score half score less than you thought. So...”* | Thinner than we thought by eye | By eye is not enough | They are usually thinner than you perceive by eye | Driver |
| *“It very much depends on the breed of your cows as well. I’ve got a mixture of cows, some are dairy bred, and you would never, you will not get fours and fives with them regardless. It’s just not….”*  *“But it will also be certain breeds. Breeds is the biggest problem we have in Scotland. Different types of breeds. Different bulls. So different sizes”.* | More to do with the breed, calf size, or the individual cow than condition scoring  Some breeds & some individuals are just harder to keep weight on or off | It is more to do with the breed of cow | Some breeds & individuals are just harder to keep weight off or on | Barrier |
| *“Just a way to manage their condition more efficiently that transition at calving definitely pays off”.*  *[Survey question: Please write below any other benefits you feel that body condition scoring can bring if these have not been covered above]*  *“Can help improve colostrum. In 6 weeks leading up to calving”*  *“Easy calving”. “Fertility”.*  *“You can pick them up quicker [the thin cows] if you put your hands on. Unfortunately, you have not got them in to do that”.* | Can find our thin cows easier by hand than by eye  Helps with issues associated with calving | Helps find thin cows | Seeing the benefits: calving ease & identifying thin cows | Driver |
| *“It would be good to learn from the model”.*  *“Need more practice”.* | It would be good to know more.  Need some practice | We would like to learn how | We would like to learn | Driver |
| *“Yeah, yeah. especially the groups from spring calving, you can't split that up”.*  *“When you start condition scoring you end up with a situation where you are having to swap them around between groups”.*  *“The problem is you have some together then you are having to shift them around after condition scoring”.* | Avoiding the grouping issues  Grouping just is not possible | Causes more issues than it resolves | No point BCS as putting them into groups causes more problems | Barrier |
| *“If there are too many groups in terms of condition score. and can't house them accordingly then there is no point”.*  *“Grouping/pen size and trying to make space”*  *“Fat ones or thin ones. Unless you got pens all over the place, you’ve got a nightmare”.*  *“You certainly don’t have enough pen space”.* | BCS/grouping pointless if you don’t have the space/pens  Going to have groups of cows all over the place  Pointless as we don’t have the room | No point if there is no space. | No point as we don’t have the space or the resources to split them into their groups | Barrier |
| *“Not accessible because they’re out at grass all year round”.*  *“My question to you is, how do you manage, how do you score them when they are at grass just now? Not everyone has a wonderful summer”?*  *“If you are out in the field, and you are trying to condition score when you are out in the field. What chance have you got at a hand?”* | Constrained by the physical environment & by resources  Can’t do by hand if they are out in the field | Not possible to BCS as they are in the field | We can’t BCS when they are out in the field; it is just not possible | Barrier |
| *“Once you are short of grass, there’s not a lot you can do”.*  *“But I'm starting to wonder if its more and more to do with the summer. We've just recently had our best calving ever . And I really don't think it's anything we did. But I think it's just more and more sun”* | It’s the climate and the grass  Better weather and grass (feeding)  It’s less about what we do (i.e., BCS) | It is just the weather that decides | It’s more to do with the weather or how much grass you have | Barrier |
| *“You talk about conditioning pretty uniformly throughout the year. But obviously the cows are going to lose condition and especially when their calves are growing. And usually you'll find in our herd anyway, that your your best cow will lose those conditions, putting their all into their calves. That shouldn't we be judging them too harshly”.*  *“If there are a couple of cows down there on the other side where they have produced one of the biggest calves and they’re weaning and that’s what’s happened”.* | Weight loss can just be the result of being a “good mother”.  A thin cow is what you will see after the mother has raised her calf, not because the farmer has failed to BCS.  Weight gain and loss is natural | A thin mum is a good mum | Weight loss is just the natural consequence of feeding the calf | Barrier |

**File C.**

**The exact instructions provided to reviewers in stage 2 of the BCW intervention mapping i.e. the TDF mapping.**

***Background to the study***

*A group of animal welfare scientists are interested in why farmers do not commonly use their hand when engaging in body condition scoring (BCS) of their cattle i.e., putting their hand on each cow to identify if she is under or overweight. Instead, most farmers use their eye to BCS, and many do not BCS as routine. Failing to closely monitor cow’s weight can have an impact on the cow’s (and her calf’s) welfare and so animal welfare scientists would like to identify ways in which we might encourage more farmers to engage in more BCS by hand, which has been identified as more accurate than BCS by eye. To do this, we need to understand the barriers and drivers of this target behaviour.*

***Instructions to reviewers:***

*You have been approached to help with this study because of your expertise in human behaviour theory. We need psychologists to review the data that we gathered from a series of focus groups and identify what human behaviour, or human cognition the data reflect. Our aim is to apply the findings from this part of the study to a human behaviour change model to later develop an effective intervention. The data that we have collected has gone through an initial qualitative content analysis to identify specific barriers and drivers of the target behaviour i.e. routinely body condition scoring cattle by hand.*

*To develop an intervention that might encourage farmers to engage in this behaviour requires us to identify what the key barriers and drivers are and apply our findings to a Human Behaviour Change model. The behaviour change model that we have chosen for our study is the Behaviour Change Wheel (BCW: Michie et al., 2014). The BCW is broken down into the COM-B hub (green hub) and the Theoretical Domains Framework (yellow hub), as illustrated below.*

Note: It is the yellow hub that we would like you to map the barriers & drivers on to


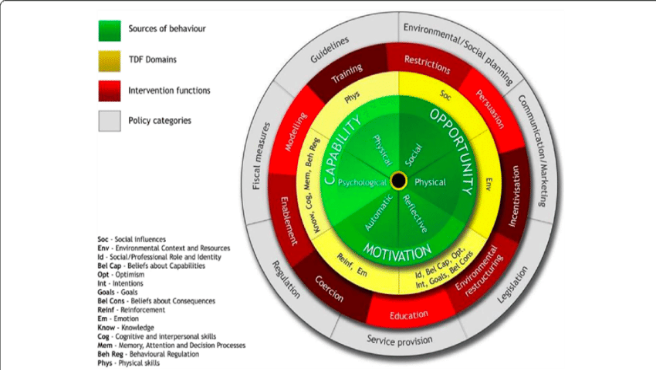


***Supplementary Figure S1:*** *The Behaviour Change Wheel showing the COM-B (green) and the Theoretical Domains Framework (yellow) (adapted from Michie et al, 2014).*

***Background***

*As previously noted, to develop an effective intervention, it is important to begin with a model or framework of behaviour (Atkins et al., 2017). The model or framework should therefore be able to identify all the mechanisms that may be involved in change, including those that are internal (psychological, physical, & motivational) and those that involve changes to the external environment (i.e., social, and material). Theories of behaviour are therefore integral to our understanding of behaviour and behaviour change. The Theoretical Domains Framework, which is provided alongside these instructions (see Table 2), was initially developed for implementation research to identify the key influences on health professionals (e.g., doctors) and patients’ (e.g., stroke survivors) behaviours. The TDF was therefore designed to provide a theoretical lens through which to view cognitive, affective, social, and environmental influences on behaviour (Atkins et al., 2017).*

*In summary, the TDF is an integrated theoretical framework synthesised from 128 constructs (e.g. schemas, attention, skills, knowledge, social norms), from 33 theories. The specific theories were identified by intervention researchers to be most relevant to questions of intervention and behaviour change (e.g., Transtheoretical Model of Change, Elaboration Likelihood Model, Health Belief Model, Self-Determination theory). Through a process of validation, 14 domains underpinned by psychological theory were identified. The 14 validated domains are: (1) knowledge, (2) skills, (3) social/professional role and identity, (4) beliefs about capabilities, (5) optimism, (6) beliefs about consequences, (7) reinforcement, (8) intentions, (9) goals, (10) memory, attention and decision processes, (11) environmental context and resources, (12) social influences, (13) emotion and (14) behavioural regulation. The domains include individual-level or internal factors, such as knowledge and skills, external or social factors such as group influence, and environment and resource factors such as time and money (Atkins et al., 2017)*

*As a psychologist, your knowledge of human behaviour and human behaviour theory places you in the ideal position to offer your insights in terms of what specific domains the farmer’s beliefs, attitudes, experiences, perceptions, insights and so forth, might fall under.*

*For example, a farmer stating that they don’t have the time or the space to BCS by hand, could be placed under domain 11:* ***Environmental Context & Resources*** *and the specific construct could be identified as:* ***Resources/Material Resources.***

*Another example is that if a farmer states that they do not BCS by hand because they do not know how to do it then this might fall under domain 1:* ***Knowledge*** *and the specific construct could be* ***Procedural knowledge i.e.,*** ***Knowing how to do something.***

*In table below (i.e. the response template) you will find the themes/categories that we identified from coding the farmers’ beliefs, attitudes, perceptions etc. These themes or categories were the result of a qualitative content analysis whereby we looked for specific drivers and barriers of BCS by hand over eye. We have included some of the quotes that were coded against each of the themes to provide you with more context.*

***What would we like you to do?***

*Pease refer to the tables provided in your additional documents, which consists of the COM-B descriptions and the TDF domains and constructs. Referring to this table please identify which domain and specific constructs the theme/category would map on to, as noted in our previous examples. Note, you do not need to map on to the COM-B components as these will naturally distil onto the TDF domains and constructs. One of the researchers will do this mapping later.*

*If you are not certain about which TDF domain/construct the category/theme maps in to then please just state so in that column. If you are of the opinion the theme could be mapped on to more than one domain or more than one construct, then please note that as we can discuss these issues later to come to some consensus. Please write all your decisions in the TDF Domain & Constructs columns. If you have any questions, please just get in touch.*

The Response Template for Mapping the Categories/Themes (& their Respective Quotes) onto the TDF domains & constructs:

| **Quotes** | **Theme/Category** | **TDF Domain** | **Construct(s)** |
| --- | --- | --- | --- |
| *e.g. “What's the fascination with getting at the side of the cow and poking about?”* | e.g. What is the actual point? |  |  |

**File D.**

**Supplementary Table S2. Reviewers' TDF mappings with agreements/disagreements.**

| **Categories/Theme** |  | **TDF Domains** | **TDF**  **Construct(s)** |
| --- | --- | --- | --- |
| What is the actual point? (B) | R1. | Beliefs about Consequences | Outcome Expectancies |
|  | R2. | Beliefs about Consequences | Outcome Expectancies |
|  | R3. | Beliefs about Consequences | Outcome Expectancies |
| ***Reviewer Agreement*** | | **3 out of 3** | **3 out of 3** |
| No point condition scoring pre-calving. (B) | R1. | Skills | Procedural Skills |
|  | R2. | Beliefs about Consequences | Outcome Expectancies |
|  | R3. | Beliefs about Consequences | Outcome Expectancies |
| ***Reviewer Agreement*** | | **2 out of 3** | **2 out of 3** |
| Uncertainty about how to body condition score by hand. (B) | R1. | Beliefs about Capabilities | Perceived competence |
|  | R2. | Knowledge | Procedural Knowledge |
|  | R3. | Knowledge | Procedural Knowledge |
| ***Reviewer Agreement*** | | **2 out of 3** | **2 out of 3** |
| Not necessary because we are all experienced farmers; we know our cows. (B) | R1. | Beliefs about Capabilities | Professional Confidence |
|  | R2. | Beliefs about Capabilities | Self Confidence |
|  | R3. | Beliefs about Capabilities | Professional Confidence |
| ***Reviewer Agreement*** | | **3 out of 3** | **2 out of 3** |
| They are usually fatter than you perceive by eye. (B) | R1. | Beliefs about Capabilities | Professional Confidence |
|  | R2. | Reinforcement | Improbable Rewards |
|  | R3. | Reinforcement | Improbable Rewards |
| ***Reviewer Agreement*** | | **2 out of 3** | **2 out of 3** |
| They are usually thinner than you perceive by eye.  (D) | R1. | Beliefs about Capabilities | Professional Confidence |
|  | R2. | Reinforcement | Rewards |
|  | R3. | Beliefs about Capabilities | Professional Confidence |
| ***Reviewer Agreement*** | | **2 out of 3** | **2 out of 3** |
| Some breeds & individuals are just harder to keep weight off or on. (B) | R1. | Environmental Context & Resources | Person x Environment |
|  | R2. | Beliefs about Capabilities | Self-efficacy |
|  | R3. | Beliefs about Capabilities | Self-efficacy |
| ***Reviewer Agreement*** | | **2 out of 3** | **2 out of 3** |
| Seeing the benefits of BCS by hand: calving ease & identifying thin cows. (D) | R1. | Beliefs about Consequences | Outcome Expectancies |
|  | R2. | Reinforcement | Rewards |
|  | R3. | Beliefs about Consequences | Outcome Expectancies |
| ***Reviewer Agreement*** | | **2 out of 3** | **2 out of 3** |
| We would like to learn. (D) | R1. | Behavioural Regulation | Action Planning |
|  | R2. | Skills | Practice Skill Development |
|  | R3. | Behavioural Regulation | Action Planning |
| ***Reviewer Agreement*** | | **2 out of 3** | **2 out of 3** |
| No point as putting them into groups causes more problems. (B) | R1. | Beliefs about Consequences | Outcome Expectancies |
|  | R2. | Beliefs about Consequences | Anticipated regret |
|  | R3. | Beliefs about Consequences | Outcome Expectancies |
| ***Reviewer Agreement*** |  | **3 out of 3** | **2 out of 3** |
| No point as we don’t have the space or the resources to split them into their groups. (B) | R1. | Beliefs about Consequences | Outcome Expectancies |
|  | R2. | Environmental Context & Resources | Resources/Material Resources |
|  | R3. | Environmental Context & Resources | Resources/Material Resources |
| ***Reviewer Agreement*** |  | **2 out of 3** | **2 out of 3** |
| We cannot BCS when they are out in the field; it is just not possible. (B) | R1. | Environmental Context & Resources | Person x Environment |
|  | R2. | Environmental Context & Resources | Person x Environment |
|  | R3. | Environmental Context & Resources | Person x Environment |
| ***Reviewer Agreement*** | | **3 out of 3** | **3 out of 3** |
| It’s more to do with the weather or how much grass you have. (B). | R1. | Environmental Context & Resources | Person x Environment |
|  | R2. | Environmental Context & Resources | Resources/Material Resources |
|  | R3. | Environmental Context & Resources | Resources/Material Resources |
| ***Reviewer Agreement*** | | **3 out of 3** | **2 out of 3** |
| Weight loss is just the natural consequence of feeding the calf. (B) | R1. | Beliefs in Capabilities | Self-Efficacy |
|  | R2. | Beliefs about Consequences | Outcome Expectancies |
|  | R3. | Beliefs about Consequences | Outcome Expectancies |
| ***Reviewer Agreement*** | | **2 out of 3** | **2 out of 3** |

**File E.**

**Mapping the barriers and drivers onto the COM-B model.**

**COM-B: Psychological Capability:**

**Uncertainty about how to BCS. (B)**

**COM-B: Reflective Motivation:**

What is the actual point? (B)

No point condition scoring pre-calving. (B)

We know our cows. (B)

Some breeds & individuals are just harder to keep weight off or on. (B)

Putting them into groups causes more problems. (B)

Weight loss is just natural. (B)

**COM-B: Physical Opportunity**

**No point as we don’t have the space/resources to split them into their groups. (B)**

**We cannot BCS when they are out in the field; it is just not possible. (B)**

**Less about what we do & more to do with the weather or how much grass you have. (B).**

**COM-B: Reflective Motivation:**

Usually thinner than you perceive by eye. (D)

BCS helps calving ease & identifying thin cows. (D)

**COM-B: Psychological Capability:**

**We would like to learn. (D)**

DRIVER

DRIVER

Barriers

**Supplementary Figure S2. An illustration of how the farmers’ beliefs/opinions mapped on to the COM-B components.**

COM-B: Capability, Opportunity and Motivation components of behaviour.

(B) denotes a barrier to body condition scoring by hand and (D) denotes a driver to body condition scoring by hand.

**File F.**

**Supplementary Table S3. Linking the intervention functions and TDF domains to their potential and most frequently used Behaviour Change Techniques (BCTs) for promoting body condition scoring.**

|  |  | **Most frequently used BCTs** |
| --- | --- | --- |
| **Intervention functions:** | |  |
| Education | | **- Information about social & environmental consequences**  **- Information about health (animal) consequences**  **- Feedback on behaviour or on outcome(s) of the behaviour**  - **Prompts/Cues**  - **Self-monitoring of behaviour or outcomes of behaviour** |
| Enablement | | - Social support (unspecified or practical)  - Goal setting (behaviour or outcome)  **- Adding objects to the environment**  - Problem solving  - Action planning*****  - **Self-monitoring of behaviour or outcomes of behaviour**  - **Restructuring the physical environment**  - Review behaviour or outcome goal(s) |
| Training | | - **Demonstration of the behaviour**  - Instruction on how to perform a behaviour*  - **Feedback on the behaviour or outcome(s) of the behaviour**  - **Self-monitoring of behaviour or outcomes of behaviour**  - Behavioural practice/rehearsal* |
| Modelling | | **- Demonstration of the behaviour** |
| Persuasion | | - Credible source*  - **Information about social & environmental consequences**  - **Information about health (animal) consequences**  **- Feedback on behaviour or outcome(s) of the behaviour** |
| Environmental Restructuring | | **- Adding objects to the environment**  **- Prompts/Cues**  **- Restructuring the physical environment** |
| **TDF Domains:** | |  |
| Knowledge (*Procedural Knowledge*) | | - **Information about health (animal) consequences**  - Biofeedback  - Antecedents  **- Feedback on behaviour or outcome(s) of the behaviour** |
| Behavioural regulation (*Action planning*) | | **- Self-monitoring of behaviour or outcomes of behaviour** |
| Beliefs about Consequences (*Outcome expectancies*) | | - Emotional consequences  - Salience of consequences  - Covert sensitisation  - Anticipated regret  - **Information about social & environmental consequences**  - Comparative imagining of future outcomes  - Vicarious reinforcement  - Threat  - Pros & cons  - Covert conditioning |

*Notes:*

- The specific TDF constructs for each respective TDF domain are in parentheses and *italicised*.
- Overlap between the BCTs that have been identified as effective for both the intervention functions and the TDF domains are in **bold.**
- Denotes the BCTs not identified more than once across the interventions and TDF domains but were identified by the researchers as relevant and important BCTs for our specific target group and target behaviour.
- TDF mappings onto their respective BCTs guided by Cane *et al*., (2015) and Michie *et al*., (2011, 2013).

References

**Ahuvia A 2001** Traditional, interpretive, and reception based content analyses: Improving the ability of content analysis to address issues of pragmatic and theoretical concern. *Social Indicators Research* **54**(2): 139–172. <https://doi.org/10.1023/A:1011087813505>

**Lumivero 2023** NVivo, Version 14. [https://www.lumivero.com](https://www.lumivero.com/).

**Michie S, van Stralen MM and West R 2011** The behaviour change wheel: A new method for characterising and designing behaviour change interventions. *Implementation Science* **6**(1). <https://doi.org/10.1186/1748-5908-6-42>.
